# MODS-Wayne, a Colorimetric Adaptation of the Microscopic-Observation Drug Susceptibility (MODS) Assay for Detection of *Mycobacterium tuberculosis* Pyrazinamide Resistance from Sputum Samples

**DOI:** 10.1128/JCM.01162-18

**Published:** 2019-01-30

**Authors:** Roberto Alcántara, Patricia Fuentes, Ricardo Antiparra, Marco Santos, Robert H. Gilman, Daniela E. Kirwan, Mirko Zimic, Patricia Sheen

**Affiliations:** a Laboratorio de Bioinformática y Biología Molecular, Facultad de Ciencias y Filosofía, Universidad Peruana Cayetano Heredia, Lima, Peru; b Department of International Health, Johns Hopkins Bloomberg School of Public Health, Baltimore, Maryland, USA; c Institute for Infection and Immunity, St. George’s, University of London, London, United Kingdom; Carter BloodCare & Baylor University Medical Center

**Keywords:** MODS, pyrazinamide, pyrazinoic acid, sputum, tuberculosis

## Abstract

Although pyrazinamide (PZA) is a key component of first- and second-line tuberculosis treatment regimens, there is no gold standard to determine PZA resistance. Approximately 50% of multidrug-resistant tuberculosis (MDR-TB) and over 90% of extensively drug-resistant tuberculosis (XDR-TB) strains are also PZA resistant. *pncA* sequencing is the endorsed test to evaluate PZA susceptibility. However, molecular methods have limitations for their wide application. In this study, we standardized and evaluated a new method, MODS-Wayne, to determine PZA resistance. MODS-Wayne is based on the detection of pyrazinoic acid, the hydrolysis product of PZA, directly in the supernatant of sputum cultures by detecting a color change following the addition of 10% ferrous ammonium sulfate. Using a PZA concentration of 800 µg/ml, sensitivity and specificity were evaluated at three different periods of incubation (reading 1, reading 2, and reading 3) using a composite reference standard (MGIT-PZA, *pncA* sequencing, and the classic Wayne test). MODS-Wayne was able to detect PZA resistance, with a sensitivity and specificity of 92.7% and 99.3%, respectively, at reading 3. MODS-Wayne had an agreement of 93.8% and a kappa index of 0.79 compared to the classic Wayne test, an agreement of 95.3% and kappa index of 0.86 compared to MGIT-PZA, and an agreement of 96.9% and kappa index of 0.90 compared to *pncA* sequencing. In conclusion, MODS-Wayne is a simple, fast, accurate, and inexpensive approach to detect PZA resistance, making this an attractive assay especially for low-resource countries, where TB is a major public health problem.

## Abstract (precis)

Although pyrazinamide (PZA) is a key component of first- and second-line tuberculosis treatment regimens, there is no gold standard to determine PZA resistance. Approximately 50% of multidrug-resistant tuberculosis (MDR-TB) and over 90% of extensively drug-resistant tuberculosis (XDR-TB) strains are also PZA resistant.

## INTRODUCTION

Tuberculosis (TB) is still an endemic disease in low-income countries, and in 2017, there were an estimated 10 million new TB cases and 1.3 million deaths (1). One of the most important threats to TB control is the increasing rate of multidrug-resistant (MDR) and extensively drug resistant (XDR) TB cases (1). Care in selecting appropriate treatment regimens is therefore vital. Pyrazinamide (PZA) is the only drug with different activity *in vivo* and *in vitro* (2). Its importance is based on its *in vivo* sterilizing effect that permits killing of persistent bacilli (3). PZA is frequently used in both first- and second-line treatment regimens (1, 4, 5). The incorporation of PZA into TB treatment regimens in the 1960s enabled the duration of first-line treatment to be reduced from 9 to 12 months to 6 months (6).

Despite its clinical importance, its mechanism of action and of resistance remain unknown (7). Briefly, PZA is a prodrug that enters the bacilli by passive diffusion. In the cytosol, it is hydrolyzed into pyrazinoic acid (POA), the active molecule against M. tuberculosis, by a reaction catalyzed by the pyrazinamidase (PZase) enzyme. POA is then expelled by an efflux system into the acidic environment (pH 5.5) where it is protonated. This reenters the bacteria and releases the proton into the cytosol. Repetition of this cycle results in the accumulation of intracellular POA plus acidification of the cytoplasm, which is lethal to the mycobacteria (2, 8–10).

Although PZA is a cornerstone in TB drug regimens, microbiological drug susceptibility testing (DST) is not routinely performed due to notorious technical limitations. However, there is evidence that the empirical use of PZA in patients with PZA resistance is associated with higher mortality rates (11), and therefore, obtaining a method of establishing PZA resistance would represent a significant advantage in the management of TB patients. Technical challenges include the influence of inoculum size, long turnaround time, and poor reproducibility test (12, 13). Crucially, DST for PZA requires an acidic culture medium (5, 14, 15), but acidity inhibits M. tuberculosis growth *in vitro* (16). Current microbiological DSTs utilize a less acidic pH compensated by a higher concentration of PZA (17). For example, Bactec Mycobacterial Growth Indicator Tube 960 PZA (MGIT-PZA), the assay most recently endorsed by the WHO for this purpose, uses a pH of 6.0 and critical concentration of 100 µg/ml PZA. However, this PZA concentration is lower than that expected for a pH of 6.0, and this may account for false reporting of PZA resistance (17–19). Moreover, the reproducibility of this method is poor (20–22).

Molecular DSTs are based on the detection of mutations in the putative promoter and gene sequence of *pncA*, which encodes PZase. These mutations often impair PZase activity and are the main cause of PZA resistance (4, 20, 23). However, the sensitivity of molecular tests is varied (45.7% to 93.0%), and specificity is low (10, 20, 24, 25), as not all mutations equally affect PZase activity (4, 20, 26). In addition, the absence of mutation hot spots, low frequency of mutations, a wide repertory of *pncA* gene mutations, and the high cost of sequencing limit their use (4, 27).

The classic Wayne test is a biochemical colorimetric test. It is inexpensive and simple to perform and uses an agar culture medium containing 100 µg/ml PZA at pH 6.4 to 6.8 (28, 29). PZA-susceptible strains release POA near neutral pH, and in the presence of ferrous ammonium sulfate (SAF), this produces a pink complex that can be visualized (reported as positive). The absence of a color change indicates the absence of POA, and therefore, PZA resistance (reported as negative) (28). The classic Wayne test has a sensitivity of 75.6 to 95.7% and specificity of 88.7 to 97% (8, 14, 29–31). However, a long turnaround time due to very low mycobacterial growth rates, the requirement of a large inoculum size, and subjective interpretation make this assay impractical for routine clinical use (8, 29, 30).

More recently, a quantitative variant of the Wayne test has been reported (8) in which the concentration of POA produced by M. tuberculosis in citrate buffer at pH 7.0 is determined spectrophotometrically. Sensitivity and specificity were high (96.0% and 97.4%, respectively), but this assay also suffers from technical restrictions.

The microscopic-observation drug susceptibility (MODS) assay can accurately diagnose TB and MDR-TB in liquid culture medium after 5 to 21 days (32, 33). It has several advantages, as follows: it can be performed directly from sputum, the cost is low, little additional equipment or training is required, and the turnaround time is short (32–36). The MODS assay has both a sensitivity and specificity approaching 100% and very good agreement with MGIT for the detection of rifampin and isoniazid susceptibility. Most recently, the MODS assay has been extended to accurately perform DST for second-line drugs (capreomycin, ciprofloxacin, cycloserine, ethambutol, ethionamide, kanamycin, *para*-aminosalicylic acid, amikacin, ofloxacin, moxifloxacin, and streptomycin) (33, 37), but susceptibility testing for PZA has not yet been achieved.

Here, we present for the first time a colorimetric-qualitative adaptation of the MODS assay to determine PZA resistance directly from sputum samples. Because of the lack of a reference method test, the results of this assay have been compared to a composite reference standard (13, 38) composed of results of MGIT-PZA, *pncA* sequencing, and the classic Wayne test to estimate sensitivity and specificity.

## MATERIALS AND METHODS

### Setting and samples.

For this study, 193 sputum samples were selected from a larger study conducted in 2015 to 2016. The remaining samples were obtained from TB patients enrolled in the National TB Strategy treatment program at the Hospital Nacional Dos de Mayo, Lima, Peru, and from the Regional Tuberculosis Reference Laboratory, Callao, Lima, Peru. All sputum samples selected for this study were positive for TB according to the MODS assay. Given that MDR-TB isolates have a 50% chance of also being resistant to PZA (3, 14, 29), in order to select a high number of PZA-resistant sputum samples for evaluation, we selected approximately 50% of specimens that were MDR and monoresistant to isoniazid or rifampin and 50% of non-MDR specimens. Data concerning patient age, sex, and TB treatment were collected. Ethical approval was obtained from the Universidad Peruana Cayetano Heredia, Lima, Peru.

### MODS for MDR-TB detection.

Sputum samples were decontaminated according to the standardized MODS protocol (39). Briefly, 2 ml of the sample was mixed with 2 ml fresh 2% *N*-acetyl-l-cysteine-sodium hydroxide (NALC-NaOH) by vortexing and then incubated at room temperature for 15 min. Next, 10 ml phosphate buffer was added to neutralize the solution. The solution was centrifuged at 3,000 × *g* for 15 min. The pellet was resuspended in 15 ml Middlebrook 7H9 culture medium (Fisher Scientific, USA) with oleic acid-albumin-dextrose-catalase (OADC; Fisher Scientific) and polymixin-B-amphotericin-B-nalidixic-acid-trimethoprim-azlocillin (PANTA; BD, USA) enrichment.

Testing for MDR was performed in a 24-well plate following a modified MODS protocol (39). Briefly, 100 µl of 7H9 medium was dispensed into the first two wells of each column on the plate. Next, 100 µl of drug solution (final concentrations, 0.4 µg/ml isoniazid [INH], 1.0 µg/ml rifampin [RIF]) was dispensed into the next two wells of each column. Nine hundred microliters of decontaminated sample was then added to each well. The plate was placed in a resealable bag (Ziploc) and incubated at 37°C for a maximum of 21 days. The plate was read according to the MODS protocol using an inverted light microscope. A positive culture was reported when cording growth of M. tuberculosis was observed in either or both of the control wells (39). For positive samples, the INH- and RIF-containing wells were read and reported on the same day, and visualization of growth was reported as resistance to the corresponding drug.

### Composite reference standard for PZA susceptibility determination.

Given the lack of a reference method to determine PZA resistance, every M. tuberculosis isolate was processed using three different assays, MGIT-PZA, *pncA* sequencing, and the classic Wayne test. A PZA-resistant sample was defined as having resistance according to at least two of the three assays, and a PZA-susceptible sample was defined as susceptibility according to at least two of the three assays (15). To perform these tests, a pure isolate was required; an aliquot from a control well of the MODS assay was inoculated onto plates containing OADC-supplemented 7H11 medium (Fisher Scientific, USA). These plates were incubated at 37°C for 14 to 21 days, after which each plate was evaluated by microscopy to detect M. tuberculosis growth. These colonies were used in subsequent assays.

MGIT-PZA was performed according to the manufacturer’s instructions. Briefly, a suspension with turbidity equivalent to 0.5 McFarland was prepared. The suspension was diluted in physiological saline at a 1:5 and 1:10 ratio, 0.5 ml of which was added to the susceptibility test tube and control tube, respectively. In addition, PZA was added to the susceptibility test tube at a final concentration of 100 µg/ml. The tubes were incubated in a MGIT 960 system. To obtain results, the fluorescent signal obtained from the control and the PZA susceptibility tubes were compared over a period of 4 to 21 days.

For *pncA* sequencing, one full loop of M. tuberculosis culture was suspended in 500 µl of Tris-EDTA buffer at pH 8.0 and inactivated by heating to 80°C for 30 min. Three-millimeter glass beads were added to the bacterial suspension. DNA extraction was performed using a modified protinase K-chloroform protocol, as previously described (40). PCR was performed as follows: 50 µl PCR master mix contained 1× PCR buffer (Thermo Scientific, USA), 2.5 µM dinucleoside triphosphates (dNTPs), 1.5 mM MgCl_2_ (Thermo Scientific), 0.5 µM primer P1 (5′-GTCGGTCATGTTCGCATCG-3′; from 105 bp upstream of *pncA*) and primer P6 (5′-GCTTTGCGGCGAGCGCTCCCA-3′; from 60 bp downstream of *pncA*) (41), and 0.03 U/µl *Taq* polymerase (Thermo Scientific). The PCR cycling parameters were set to one cycle of 94°C for 4 min, followed by 30 cycles of 94°C for 45 s, 58°C for 60 s, and 72°C for 60 s, with a final step of 94°C for 5 min. The 720-bp amplification products were sequenced using the same primers that were used for PCR. The presence of mutations in both the *pncA* gene and the putative promoter was evaluated by pairwise sequence alignment with the nucleotide sequence of the M. tuberculosis H37Rv reference strain (NCBI RefSeq accession no. NC_000962.3). Identified mutations were compared with those reported in the Tuberculosis Drug Resistance Mutation Database (42).

The classic Wayne test was performed as described previously (28). Briefly, a heavy loopful of 21-day M. tuberculosis culture was inoculated onto Dubos culture medium. The tubes were incubated at 37°C for 7 days. For each test round, positive (H37Rv) and negative (DM097) controls were included. For the test reading, 1 ml 1% SAF (Sigma-Aldrich, USA) was added, and the tubes were inspected for the presence of a pink band. Where this was not seen, the tubes were incubated at 4°C for an additional 4 h and then read again, and this was reported as the final result; the presence of a pink band indicated a susceptible strain, and the absence of color indicated a resistant strain.

### Acid-fast bacillus smear.

In order to explore a potential effect of the bacillary load in sputum with the MODS-Wayne assay, each sample was evaluated by smear microscopy using Ziehl-Neelsen staining. The procedure was performed and the slide interpreted according to standard techniques (43).

### MODS-Wayne assay.

A variant of the MODS assay (MODS-Wayne) was standardized to determine PZA resistance directly from decontaminated sputum samples. This variant utilizes a Middlebrook 7H9 culture broth enriched with OADC and PANTA (pH 6.8) that favors the growth of M. tuberculosis isolates in culture. Unlike traditional PZA susceptibility tests, MODS-Wayne does not require an acidic pH because the goal of the assay is to detect the production of POA from PZA metabolism and not to evaluate PZA bacterial killing. The neutral pH permits the POA to accumulate in the supernatant where it can be detected using SAF.

**FIG 1:**
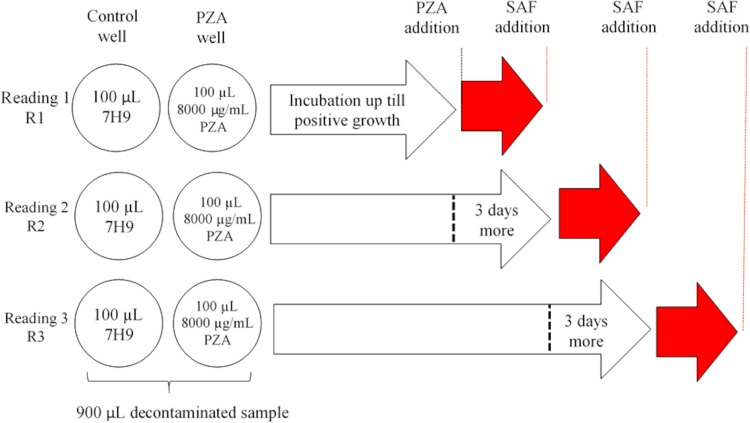
MODS-Wayne plate design. Three samples were evaluated in each plate. One control well was included for every sample for each reading (R1, R2, and R3). Red arrows represent the 3 days of incubation after pyrazinamide (PZA) addition. Ferrous ammonium sulfate (SAF) was added after the incubation with PZA.

**FIG 2:**
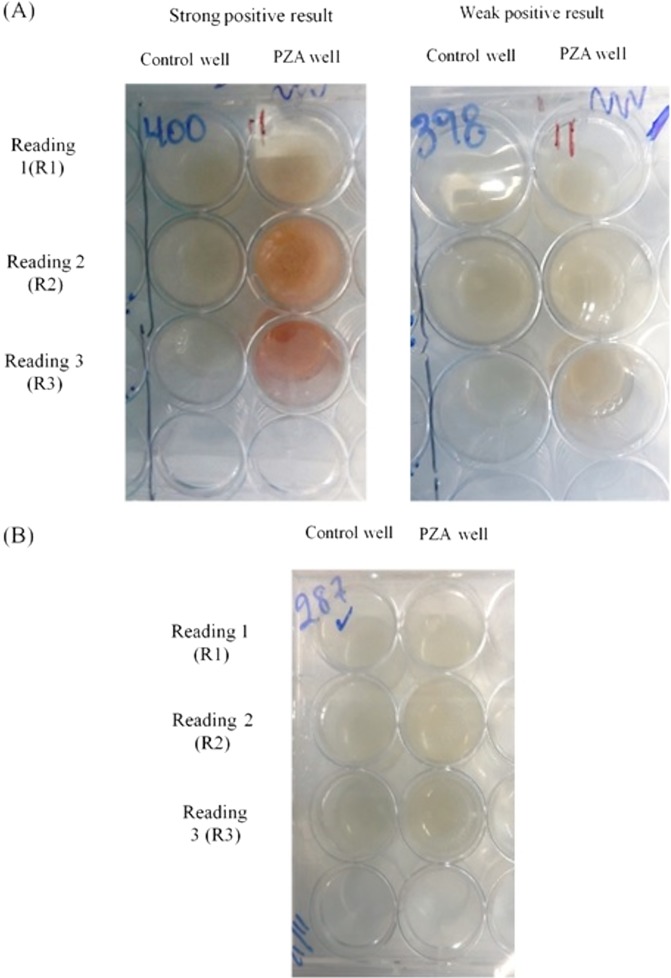
MODS-Wayne results. The intensity of the color is enough to allow discrimination between a positive (presence of pink color) result (A), and a negative (absence of pink color) result (B), indicating pyrazinamide (PZA) susceptibility and resistance, respectively.

Each decontaminated sample was cultured in 6 wells in a 24-well plate. Nine hundred microliters of the decontaminated sample was seeded per well. Three wells corresponded to control wells, and the other 3 wells were designated for PZA addition. As the test is performed using decontaminated sample, it is possible that the number of viable bacilli, or bacillus viability, could be reduced by stress during the decontamination process; in order to increase the number of bacilli and/or to improve the viability of stressed bacteria, the plate was first incubated at 37°C with no drug. The plate reading commenced on day 5 and was then performed every 2 days until day 21, according to the standard MODS protocol (39). When at least two microcolonies were observed in the control wells, 100 µl PZA (8,000 µg/ml) (Sigma-Aldrich, USA) was added to one of the PZA wells (final concentration, 800 µg/ml). After 3 days and 6 days, the same volume of PZA was added to one of the remaining PZA wells. Based on previous results, in order to facilitate the production of a detectable amount of POA by a Wayne reaction, after the addition of PZA, the samples were incubated for 3 days, during which time the bacilli were able to produce POA. The nonacidic pH (6.8) prevented the lethal effect of the drug and thus the inhibition of growth, and these conditions would be expected to maximize the production and accumulation of POA, the pivotal biomarker of PZA resistance. At the end of this additional period (reported as reading 1 [R1], reading 2 [R2], and reading 3 [R3]) (Fig. 1), 100 µl of 10% ferrous ammonium sulfate (Sigma, USA) was added to the corresponding control and PZA wells, and the color was registered immediately. A positive result was reported when a pink color was observed in the PZA well but not in the control well, and a negative result was reported when no color was observed in either the control well or the PZA well (Fig. 2A and B). Additionally, the level of growth on culture was reported in one of four categories according the degree of aggregation by microscopy, high growth, regular growth, low growth, and microcolonies, to evaluate any possible influence on the MODS-Wayne assay.

## RESULTS

Agreement between the dichotomous MODS-Wayne results and the composite reference standard for each of the three drug incubation periods was estimated using the Kappa statistic in a 2-by-2 contingency table. The potential effect of bacillary load or level of growth on the MODS-Wayne results was evaluated using Fisher’s exact test. For results with a positive association, a proportion test was performed to evaluate significant differences. The sensitivity and specificity (95% confidence interval [CI]) of the MODS-Wayne assay compared to those of the consensus reference test were estimated for each drug incubation period. McNemar’s test was used to determine significant differences between sensitivities and specificities among MODS-Wayne results. All statistical tests were reported with 95% confidence intervals and were performed using Stata version 14.0.

### Study population.

Sputum samples were obtained from 193 patients. The descriptive statistics for the study population are shown in Table 1. Data were missing for the following fields: TB treatment (79/193 [40.9%]), age (43/193 [22.3%]), and sex (22/193 [11.4%]).

**TABLE 1:** Description of patients from whom sputum samples were obtained^*a*^

Characteristic	No. (%)
Patients	
Callao reference lab	162 (83.9)
Hospital Nacional Dos de Mayo	31 (16.1)
Sex	
Female	59 (30.1)
Male	113 (58.6)
Data not available	22 (11.4)
Age (yr)	
13–22.3	33 (17.1)
22.3–31.5	30 (15.5)
31.5–40.8	27 (13.9)
40.8–50	23 (11.9)
50–59.3	19 (9.8)
59.3–68.5	8 (4.1)
68.5–77.8	6 (3.1)
77.8–87	4 (2.1)
Data not available	43 (22.3)
TB treatment	
Never treated	93 (48.2)
Previous or ongoing treatment	21 (10.9)
Data not available	79 (40.9)

a All samples met the following criteria: minimum volume of 2 ml and positive acid-fast microscopy results (health center’s results).

Of the 193 sputum samples collected, 101 (52.3%) were pansusceptible according to the MODS assay. Ninety-two samples (47.7%) had growth in the INH- and/or RIF-containing wells, indicating resistance; of these, 23 samples (11.9%) were monoresistant to isoniazid, 12 samples (6.2%) were monoresistant to rifampin, and 57 samples (29.5%) were resistant to both drugs (i.e., MDR).

### Composite reference standard for PZA susceptibility determination.

According to MGIT-PZA, 147 samples (76.2%) were PZA susceptible, and 46 samples (23.8%) were resistant. According to the classic Wayne test, 162 samples (83.9%) were PZA susceptible, and 31 samples (16.1%) were resistant. At least one mutation was detected in the *pncA* gene in 51/193 samples (26.4%). Forty-seven (92.2%) of the mutations were single nucleotide polymorphisms, and four samples (2.1%) had a deletion in the *pncA* gene (nucleotides 376 to 389 or 456 to 466). SNPs were found at the putative promoter (A-11G), metal-binding site (D49N, H51R, H57L, H57R, and H71R), and the active site (D8E). The PZA susceptibility profile was given according to data reported in the Tuberculosis Drug Resistance Mutation database (42); 154 samples (79.8%) were classified as PZA susceptible by *pncA* sequencing. This group included strains with a wild-type phenotype and with K48T, P62S, and F81S mutations. The rest of the strains with mutations were reported as PZA resistant (39/193 [20.2%]) (Table 2).

**TABLE 2:** Mutations in the putative promoter and *pncA* gene reported in this study^*a*^

Genotype	Reported frequency	PZA susceptibility profile	%
Promoter (A-11G)	1	Resistant	0.5
D49N	1	Resistant	0.5
D8E	2	Resistant	1.0
F81S	2	Susceptible	1.0
H51R	10	Resistant	5.2
H57L	1	Resistant	0.5
H57R	1	Resistant	0.5
H71R	2	Resistant	1.0
I6S	2	Resistant	1.0
K48T	9	Susceptible	4.7
P62S	1	Susceptible	0.5
Q10P	2	Resistant	1.0
Q10R	13	Resistant	6.7
Δ375–389	1	Resistant	0.5
Δ456–466	3	Resistant	1.6
Wild type	142	Susceptible	73.6

a Specific mutations are reported along with the strain’s corresponding PZA susceptibility profile according to the Tuberculosis Drug Resistance Mutation Database.

**FIG 3:**
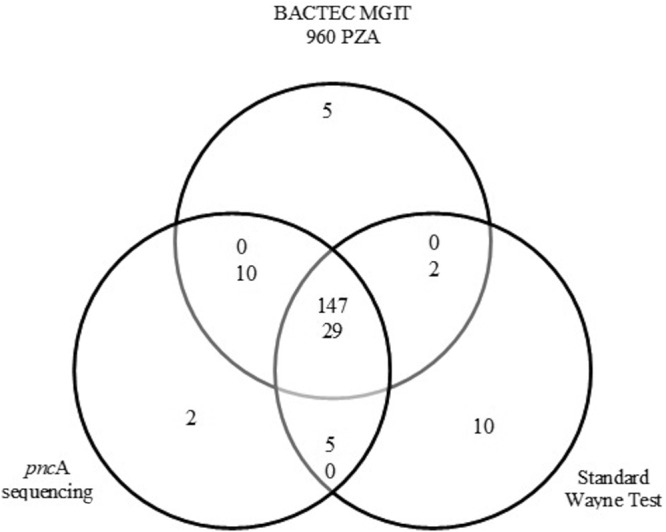
Sample distribution by PZA susceptibility profile. Distribution displayed according to the results from Bactec MGIT 960 PZA, *pncA* sequencing, and classic Wayne test. For stacked numerals, the number of PZA-susceptible isolates is given above the number of PZA-resistant isolates.

Overall, according to the composite reference standard, 152 samples were reported as PZA susceptible (78.8%), and 41 samples were reported as PZA resistant (21.2%). Among these, 147 samples (76.2%) were sensitive according to all three tests used, and 29 samples (15.0%) were resistant according to all three tests (Fig. 3). For MDR and RIF-resistant isolates, 52.6% (30/57) and 58.3% (7/12) were reported as PZA resistant by the consensus reference test, respectively. In contrast, just 4.0% (4/101) of the pansusceptible isolates were reported as PZA resistant. No PZA resistance was reported in INH-monoresistant isolates.

### Acid-fast bacillus smear.

Acid-fast smear testing was positive for 182 samples. Of these samples, 6.0% (11/182) were reported as paucibacillary (less than 1 bacillus seen per microscopic field), 22.0% (40/182) as 1+, 33.5% (61/182) as 2+, and 38.5% (70/182) as 3+; 5.7% of the samples (11/193) were smear negative. A trend between bacillary load and the positivity of MODS-Wayne at the earlier time points was observed; however, this did not reach statistical significance (Fisher’s exact test, *P* = 0.06 for R1, *P* = 0.081 for R2, and *P* = 0.514 for R3).

### MODS-Wayne performance.

The proportion of negative results (resistant pattern) decreased with increasing reading day, from 104/193 (53.9%) to 39/193 (20.2%) (Table 3). By R3, negative results (PZA resistance) were reported in 5/101 (4.95%) of the pansusceptible isolates, 27/57 (47.4%) of the MDR-TB isolates, and 7/12 (58.3%) of the RIF-resistant isolates. All INH-resistant isolates (23/23) were reported as positive (PZA susceptible).

**TABLE 3:** Susceptibility to PZA determined by the MODS-Wayne assay for each reading day^*a*^

Reading	MODS-Wayne results (no. of isolates susceptible or resistant [% of total])	
	PZA susceptible	PZA resistant
R1	89 (46.1)	104 (53.9)
R2	132 (68.4)	61 (31.6)
R3	154 (79.8)	39 (20.2)

a *n* = 193 isolates. With increasing incubation time, the assay reported an increasing proportion of PZA-susceptible isolates.

On mycobacterial culture, 50.3% of the total samples (97/193) had a high level of growth, 30.1% (58/193) had regular growth, 12.4% (24/193) had low growth, and 7.3% (14/193) had microcolonies only. Similarly to the MODS-Wayne results, the proportion of positive samples increased as reading day increased for each level of growth reported (Table 4). A significant association was observed between the level of growth and the positivity with MODS-Wayne assay at R1 (*P* = 0.045) and at R2 (*P* = 0.008) but not at R3 (*P* = 0.09).

**TABLE 4:** Proportion of samples with positive and negative results by MODS-Wayne assay per reading day^*a*^

Level of growth in culture (*n*)	No. (%) with result by reading day					
	R1		R2		R3	
	Positive	Negative	Positive	Negative	Positive	Negative
Microcolonies (14)	3 (21.4)	11 (78.6)	6 (42.9)	8 (57.1)	9 (64.3)	5 (35.7)
Low growth (24)	8 (33.3)	16 (66.7)	11 (45.8)	13 (54.2)	16 (66.7)	8 (33.3)
Regular growth (58)	25 (43.1)	33 (56.9)	43 (74.1)	15 (25.9)	50 (86.2)	8 (13.8)
High growth (97)	53 (54.6)	44 (45.4)	72 (74.2)	25 (25.8)	79 (81.4)	18 (18.6)

a An increase in the number of samples with positive results was observed along the reading days.

High and regular growth levels were associated with a higher proportion of positivity in the MODS-Wayne assay at R2 (*P* < 0.0001), but no significant association was observed for samples with low growth or microcolonies (*P* = 0.56 and 0.45, respectively). Conversely, low growth level and microcolonies were associated with a higher proportion of negative MODS-Wayne results at R1 (*P* = 0.011 and 0.0012, respectively), but no significant association was observed for samples with high and regular growth (*P* = 0.20 and 0.14, respectively).

### Accuracy measures for MODS-Wayne.

Results from each reading day were compared to the composite reference standard independently. Sensitivities (true resistant percentage) in R1, R2, and R3 were similar, ranging from 92.7% to 97.6% (*P* > 0.05). However, the specificities showed a broader range, increasing from 57.9% to 99.3% (Table 5) and with a statistically significant difference upon comparison (*P* < 0.05).

**TABLE 5:** Performance of MODS-Wayne assay compared to composite standard^*a*^

Reading day	Sensitivity		Specificity	
	% (no. of isolates/total no. of isolates)	95% CI (%)	% (no. of isolates/total no. of isolates)	95% CI (%)
R1	97.6 (40/41)	87.1–99.9	57.9 (88/152)	49.6–65.9
R2	97.6 (40/41)	87.1–99.9	86.2 (131/152)	79.7–91.2
R3	92.7 (38/41)	80.1–98.5	99.3 (151/152)	96.4–100

a Each diagnostic value was calculated with 95% CI.

As the MODS-Wayne and the classic Wayne assays obey the same principles, they were directly compared. At day 16, the sensitivity of MODS-Wayne was 93.5% and specificity was 93.8%, with an agreement and kappa index of 93.8% and 0.79, respectively (*P* = 0.001). (Data from other time points not shown.) Ten isolates were PZA resistant according to the MODS-Wayne assay but PZA susceptible according to the classic Wayne test. When their genotypes were analyzed, nine isolates had mutations associated with PZA resistance (Q10P, Q10R, H51R, and H57R), and one strain was wild type.

## DISCUSSION

This study shows for the first time that an adaptation of the MODS assay combined with the Wayne test (MODS-Wayne) can accurately determine M. tuberculosis resistance to PZA directly from AFB smear-positive sputum samples and can achieve this in a relatively short period of time compared to other PZA DSTs. In addition, it is inexpensive, simple to perform, and requires minimal training of laboratory staff. The MODS-Wayne assay is based upon the principle that POA is only produced by PZA-susceptible bacilli, and therefore, detection of POA in the supernatant of a MODS culture through visual observation of a Wayne colorimetric reaction functions as an accurate proxy for PZA sensitivity. In contrast with previous efforts to modify MODS for PZA resistance testing, in this adaptation, after sputum samples are decontaminated, the stressed M. tuberculosis bacilli are grown at a nonharmful neutral pH, in the absence of any drug until mycobacterial growth is observed. PZA is only added after this time. The neutral pH is maintained throughout. This allows amplification of the bacterial population, thereby maximizing the release of POA into the extracellular environment while preventing bacterial killing. As MODS-Wayne only evaluates the presence of POA in the supernatant, there is no need to satisfy the culture conditions, including an acidic pH, necessary to demonstrate PZA’s antibacterial activity. The advantage of using a neutral pH is that acidity inhibits the growth of M. tuberculosis and therefore reduces the test’s specificity (44). MODS-Wayne overcomes other limitations of the traditional DSTs for PZA, in that there is no requirement for an isolate to be grown first (indirect test), or for a large inoculum size (3 to 5 mg) in order to avoid false-negative results (30).

MODS-Wayne reported a median turnaround time of 18.5 days (range, 14 to 29 days) for PZA-susceptible isolates and 21 days (range, 14 to 25 days) for PZA-resistant isolates for results in R3. Although the MODS-Wayne turnaround time was higher than that reported for direct MGIT-PZA (range, 11 to 16 days), Demers et al. (45) reported that 41% (163/398) of the direct MGIT-PZA results were uninterpretable mostly due to growth failure, and most of the indirect tests (i.e., classic Wayne test) require primary isolation of the isolate first, which means that from the time when the specimen was collected until the susceptibility results were obtained, the turnaround time ranged from 18 to 95 days. Total time from sample to results is in fact much shorter for MODS-Wayne, which is a direct test.

The use of a composite standard where no good single standard exists has been described (12). Using our composite reference standard (MGIT, classic Wayne test, and *pncA* sequencing), we found that around half of the selected MDR-TB strains were PZA resistant (30/57 samples [52.6%]), but few of the selected pansusceptible strains were PZA resistant (4/101 samples [4.0%]). These findings are in agreement with rates reported by other studies (3, 6, 20).

Both the classic and the quantitative Wayne assays (8, 28) evaluate PZase activity using PZA concentrations ranging from 100 µg/ml to 400 µg/ml (21). In this study, we used a PZA concentration of 800 µg/ml in order to obtain a maximum signal without compromising specificity. This concentration was established in a previous study optimizing the assay, as it enabled us to most accurately discriminate isolates with weak-positive Wayne activity (PZA susceptible) from those with negative Wayne activity (PZA resistant) (our unpublished data). Compared to the classic Wayne test, MODS-Wayne showed an agreement and kappa index of 93.8% and 0.79, respectively. Ten isolates showed discrepant results, i.e., were PZA susceptible according to the classic Wayne assay and PZA resistant according to MODS-Wayne; nine isolates clearly have genuinely PZA-resistant phenotypes because they have mutations in *pncA* that are close to the active site (Q10P and Q10R) or affect directly the metal-binding site (H51R and H57R), both of which are associated with PZA resistance (7). Additionally, these isolates reported PZA resistance by MGIT-PZA. This discrepancy may be attributable to misinterpretation during the reading of the classic Wayne assay, as difficulty in the correct interpretation of weak-positive samples has been reported as a limitation of this test (8, 29, 30). On the contrary, the single wild-type strain that was falsely reported as PZA resistant by the MODS-Wayne may have been be due to the lack of PZA addition during the incubation period. This isolate was reported as PZA susceptible by MGIT-PZA.

Although MODS-Wayne does not evaluate *pncA* mutations, it had 96.9% agreement and a kappa index of 0.90 with *pncA* sequencing. One disadvantage of molecular methods is that interpretation of the results requires an understanding of the roles of *pncA* gene mutations and PZase activity (4). Not all mutations in the putative promoter or the *pncA* gene have been reported, and not all reported mutations are associated with PZA resistance. Nevertheless, MODS-Wayne is a phenotypic test that indirectly evaluates *pncA* mutations (i.e., mutations that affect *pncA* expression or impair PZase activity) by detecting POA, the enzymatic product of the PZase. In this case, a mutation database is not required to interpret the results. Six of 7 isolates harboring K48T maintained PZase activity and were reported as positive by MODS-Wayne and PZA susceptible by MGIT-PZA. K48T is an example of a mutation that is not associated with PZA resistance. More studies are necessary to understand the real effect of some mutations that do not impair PZase activity on PZA susceptibility.

As a phenotypic test, MODS-Wayne depends on the growth of M. tuberculosis in culture and its capacity to produce a detectable amount of POA. Our results showed that the level of growth influenced the time to positivity on MODS-Wayne, and there was also a trend in association of the bacillary load on smear microscopy with time to results, although this did not reach statistical significance. We hypothesized that a low number of PZA-susceptible bacilli can lead to a negative result in the early reading days, which can be considered false PZA resistance. This could be mitigated by using a longer duration of culture prior to the addition of PZA to the assay, which would increase the number of PZA-susceptible bacilli and give a positive MODS-Wayne result.

Our data show that the accuracy of MODS-Wayne (92% sensitivity and 99.3% specificity) is comparable to that of other tests for PZA resistance detection (MGIT, classic Wayne, and *pncA* sequencing). The MODS-Wayne assay and MGIT-PZA had an agreement and kappa index of 95.3% and 0.86, respectively. The sensitivity and specificity of the MODS-Wayne assay were optimal in R3 (92.7% and 99.3%, respectively); although sensitivity was slightly higher in R2 (97.6%), our assumption is that longer culture duration may permit a more accurate discrimination of the PZA-resistant from the PZA-susceptible isolates. This will need to be confirmed in future studies.

Finally, it is important to highlight that the MODS-Wayne assay lends itself to incorporation into the standard MODS assay. This would generate a single unique test that would be able to diagnose TB, MDR-TB, XDR-TB, and PZA resistance from the same sputum sample with high accuracy and in a relatively short time.

In conclusion, here, we describe the MODS-Wayne test, a modification of the standard MODS method that is able to determine PZA susceptibility with high accuracy and in a relatively short time. Although DST for most first- and second-line TB drugs is available, PZA susceptibility testing is not routinely performed despite widespread use of the drug because of well-described difficulties in testing its activity *in vitro*. MODS-Wayne overcomes these difficulties and also has the advantages of low cost and being easy to perform and interpret. Thus, the MODS-Wayne assay offers the real possibility of introducing routine PZA susceptibility testing in resource-constrained and/or high-TB-burden settings. This would represent a major advance in the treatment of TB patients worldwide.
